# Yeast *Yarrowia lipolytica* as a Chassis for Polyester Biodegradation—A Comparative Analysis of the Diverse Wild-Type Strains and Their Engineered Derivative That Overexpresses the LIP2 Lipase

**DOI:** 10.3390/ijms27094073

**Published:** 2026-05-01

**Authors:** Julia A. Dybka, Aleksandra M. Mirończuk

**Affiliations:** Laboratory for Biosustainability, Institute of Biology, Wrocław University of Environmental and Life Sciences, ul. Kożuchowska 5b, 51-630 Wroclaw, Poland

**Keywords:** lipase, Lip2, *Yarrowia lipolytica*, polyester, biodegradation

## Abstract

In this study we compared seven wild-type strains of *Yarrowia lipolytica* as a chassis for aliphatic polyester biodegradation. To this end, we overexpressed the native Lip2 lipase under the strong UASB16-TEF promoter and compared the capability of the engineered strains towards polycaprolactone (PCL) biodegradation. The parental strains are wild types isolated from various sources and geographical regions. Despite the fact that all belong to the *Yarrowia lipolytica* species, a high diversity among the strains was observed. This study clearly indicates that, for biotechnological applications of the engineered strains, it is crucial to select a proper host for an efficient process.

## 1. Introduction

In recent decades, the accumulation of synthetic plastic waste has grown to become one of the most critical threats to global ecosystems [1,2]. While conventional petroleum-based polymers such as polyethylene terephthalate (PET), polyethylene (PE) or polypropylene (PP) are highly resistant to environmental degradation, there is growing scientific and industrial interest in biodegradable alternatives [3]. Polycaprolactone (PCL) is a semi-crystalline aliphatic polyester which, due to its structural properties, serves as a model substrate for studying the mechanisms of the enzymatic depolymerization of plastics [4,5]. Although PCL is considered biodegradable, its hydrolysis rate in the natural environment remains limited [6], which stimulates the search for microorganisms capable of efficiently secreting depolymerizing enzymes [7].

Among microorganisms with high potential for the utilization of hydrophobic waste, the yeast *Yarrowia lipolytica* shows unique features. This species naturally inhabits environments rich in lipids and alkanes, which has led to the evolutionary development of efficient metabolic pathways for the uptake and degradation of hydrophobic compounds [8]. *Y. lipolytica* has GRAS (Generally Recognized As Safe) status and is an established platform in industrial biotechnology, widely used for the production of organic acids, enzymes, and lipids [9,10]. The biodegradation potential of these yeasts is largely based on the broad spectrum of secreted hydrolytic enzymes, including proteases, esterases, and lipases [10], which play a key role in the lipolytic system of *Y. lipolytica*. Lip2 is the main lipolytic enzyme of this organism, responsible for the hydrolysis of triacylglycerols and fatty acid esters [11]. It was shown that native lipases from *Y. lipolytica* may exhibit significant activity toward aliphatic polymers such as PCL, especially under conditions of overexpression or in strains with high secretory activity [12]. The use of the host’s native enzymatic machinery offers a clear advantage, potentially reducing the metabolic burden associated with the expression of heterologous proteins [13].

However, most genetic engineering strategies in *Y. lipolytica* are currently limited to a narrow group of model strains, such as W29 or auxotrophic strain PO1f (deficient in uracil and leucine biosynthesis; widely employed for genetic engineering) [14]. As a result, there is a clear gap in the literature regarding the influence of the genetic background of different wild isolates on polymer degradation efficiency. Wild strains often show considerable variability in secretion capacity, proteolytic profiles, and growth kinetics, which can drastically affect the outcome of biotechnological processes [15]. The aim of this study was to compare seven wild-type *Y. lipolytica* strains and their derivatives that overexpress the native *Lip2* gene. To investigate the influence of the genetic background, wild-type isolates A-101, F1-3, H222, W29, CBS2174, CBS7033 and CBS8108 were used. This work provides a comprehensive analysis of their PCL degradation capacity, including growth kinetics and activity, supplemented by an analysis of morphological changes in the film surface using scanning electron microscopy (SEM).

## 2. Results

### 2.1. Construction and Verification of Y. lipolytica Strains Overexpressing the LIP2 Gene

The first step in this study was the overexpression of native *Lip2* gene in seven different *Y. lipolytica* strains to select the most suitable chassis for polymer biodegradation. To this end, the native *Lip2* gene was cloned under the control of a constitutive hybrid promoter UAS_B16_-TEF [16], using the pAD-Lip2-H vector. The proper overexpressing cassette has been transformed into wild-type strains, and hygromycin was used as a selection marker. The integration in the gDNA was confirmed by PCR. These strains were further designated as [Strain] pAD-Lip2-H. From each strain, two biological replicates have been selected for further study. To select clones with the highest protein activity, strains were spotted into plates with PCL. As was shown before, the activity of LIP2 can be observed by a halo around the colony [5]. The clones with the widest halo were selected for further study.

### 2.2. Level of LIP2 Gene Overexpression (qRT-PCR Analysis)

**Figure 1:**
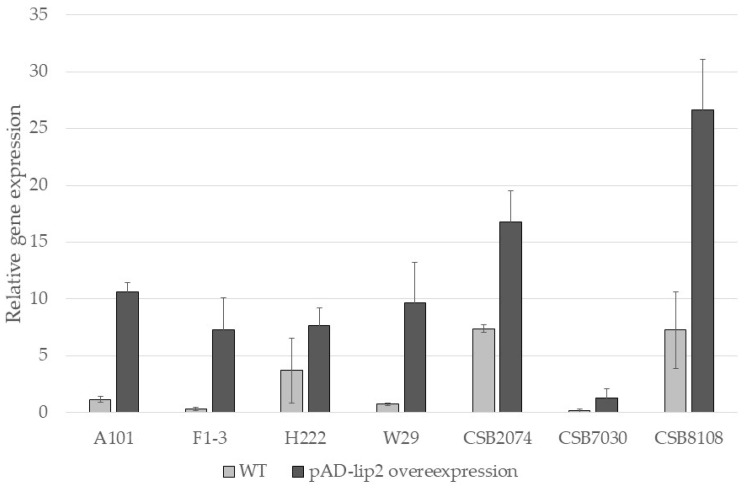
Relative quantification of RNA transcript in the wild strains and the strains overexpressing Lip2 (pAD-lip2-H) using qRT-PCR. Actin was used as a reference gene. Strains were grown in YPD medium. Samples were analyzed in triplicate, and the standard errors were estimated using CFX96 Touch Real-Time PCR Detection System (Bio-Rad Laboratories, Inc., Hercules, CA, USA).

To verify the functional overexpression of the obtained strains, we performed a qRT-PCR analysis. In this study, we compared the relative expression levels of the *Lip2* gene in wild types and the engineered strains. The results are shown in Figure 1. As the *Lip2* gene is present in the *Y. lipolytica* genome, we observed expression in all tested strains. In the wild types, LIP2 production is correlated with the presence of a hydrophobic substrate in the medium; the expression of the Lip2 gene was moderate for the wild-type strains, because all strains were grown in YPD medium. We observed elevated Lip2 expression in all engineered strains compared to their parental strains. The highest level of *Lip2* expression was observed in the engineered CBS8108 pAD-lip2-H strain. The engineered strain with the lowest level of *Lip2* expression was the CBS7033 pAD-lip2-H strain; however, the level of Lip2 expression was still higher in the engineered strain than in the parental strain, CBS7033. The qRT-PCR analysis confirmed that the *Lip2* gene was functionally overexpressed in all modified strains.

### 2.3. Growth Kinetics of Transformants

**Figure 2:**
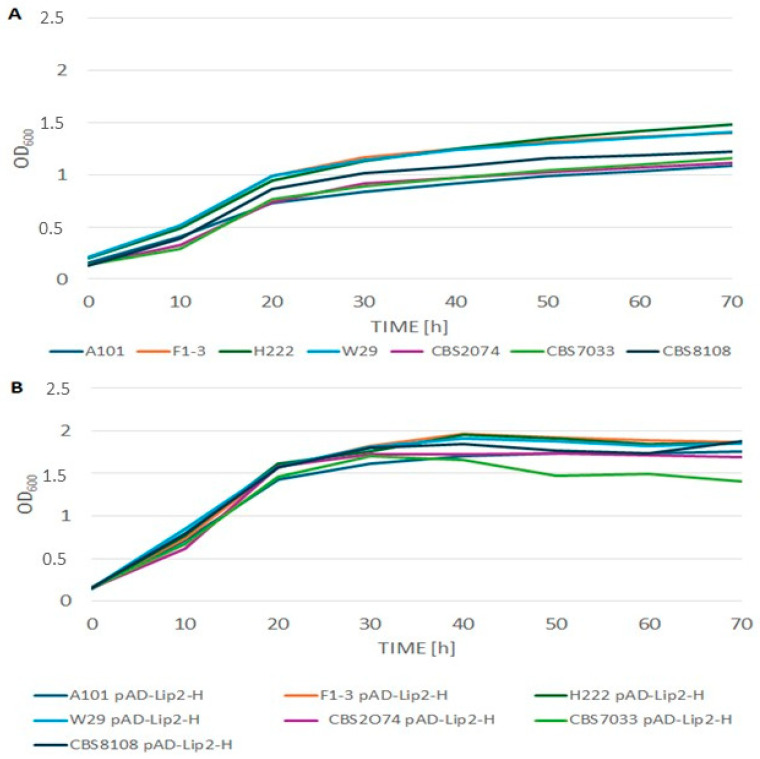
Growth kinetics of the tested *Y. lipolytica* strains in microplate culture in YPD medium. (**A**) Parental strains (control). (**B**) The engineered strains with overexpression of the LIP2 gene (pAD-Lip2-H). Optical density (OD_600_) measurements were performed automatically every 30 min. The experiment was performed in three independent biological replicates.

Since metabolic engineering and protein overexpression can place a metabolic burden on cells, the growth kinetics of the transformants were evaluated using a microplate system (Figure 2). A comparison of growth profiles showed that the presence of the pAD-Lip2-H construct, which leads to constitutive lipase production, did not have a negative effect on cell proliferation. Interestingly, the wild-type strains show some differences in their growth (Figure 2A); however, all strains achieved OD_600_ > 1.0 after 48 h of cultivation. We noticed that the engineered strains (Figure 2B) showed dynamic growth, reaching the stationary phase faster (approx. 20–30 h) and achieving higher final optical density values (OD_600_ in the range of 1.7–2.0) compared to the parental strains (OD_600_ in the range of 1.0–1.5). The exception was strain CBS7033 pAD-Lip2-H, in which a gradual decrease in optical density was observed after 40 h of cultivation, which may suggest an earlier entry into the death phase or cell autolysis. Nevertheless, the results obtained clearly demonstrate that the engineered strains retain high viability and biomass production capacity.

### 2.4. Degradation of Aliphatic Polyesters by the Wild-Type and the Engineered Y. lipolytica Strains

Following verification of the functional *Lip2* expression and growth capability the strains, we tested the biodegradation capability of the wild-type and engineered strains using PCL as a model aliphatic polyester. To this end, the strains were grown overnight at 28 °C in YPD. Consequently, the OD of the tested strains was standardized: they were spotted onto PCL plates and incubated for 24 h at 28 °C (for details, see Section 4).

**Figure 3:**
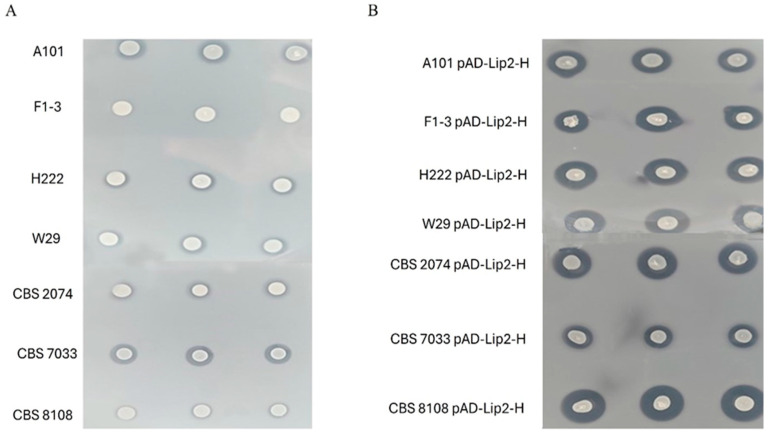
Comparison of lipolytic activity against polycaprolactone (PCL) using the spot test method. (**A**) Parental strains (wild types). (**B**) The engineered strains with overexpression of the LIP2 gene (pAD-Lip2-H). The photos show colony growth on FMM medium with the addition of 0.1% PCL emulsion.

First, we observed a significant difference between the wild-type strains (Figure 3A). The widest halo was observed for strains A101 and CBS7033, suggesting that native enzymes were highly expressed. Interestingly, qRT-PCR analysis showed moderate *Lip2* expression for CBS7033, suggesting that this strain may naturally express other lipases/hydrolases. The strain F1-3 showed the smallest halo, whereas the remaining strains showed similar sizes of halos. This finding agrees with the qRT-PCR analysis shown in Figure 1 and demonstrates that there is significant variation between *Y. lipolytica* strains, suggesting that each process should be tested in relation to the host strain. In agreement with qRT-PCR analysis, we observed an enhanced activity of the Lip2 gene in all of the engineered strains in comparison to the parental strains (Figure 3B). In accordance with the previously observed variability, the diversity between strains was noticed during this experiment. Despite the fact that A101 showed the widest halo, the overexpression of LIP2 did not significantly improve its enzymatic activity.

A similar effect was observed for the CBS7033 pAD-Lip2-H strain. Between the engineered strains, CBS8108 showed the most spectacular effect of Lip2 overexpression: the halo is the widest among all tested strains. In addition, strain F1-3 pAD-Lip2-H showed much higher activity than its parental strain, suggesting that a simple overexpression of the *Lip2* gene is sufficient for PCL biodegradation. The size of the clear zones was comparable for most of the engineered strains, suggesting that the overexpression of the *Lip2* gene effectively eliminates differences in the natural secretory efficiency of individual wild-type strains.

### 2.5. Biodegradation of PCL Films During Shake-Flask Experiments

**Figure 4:**
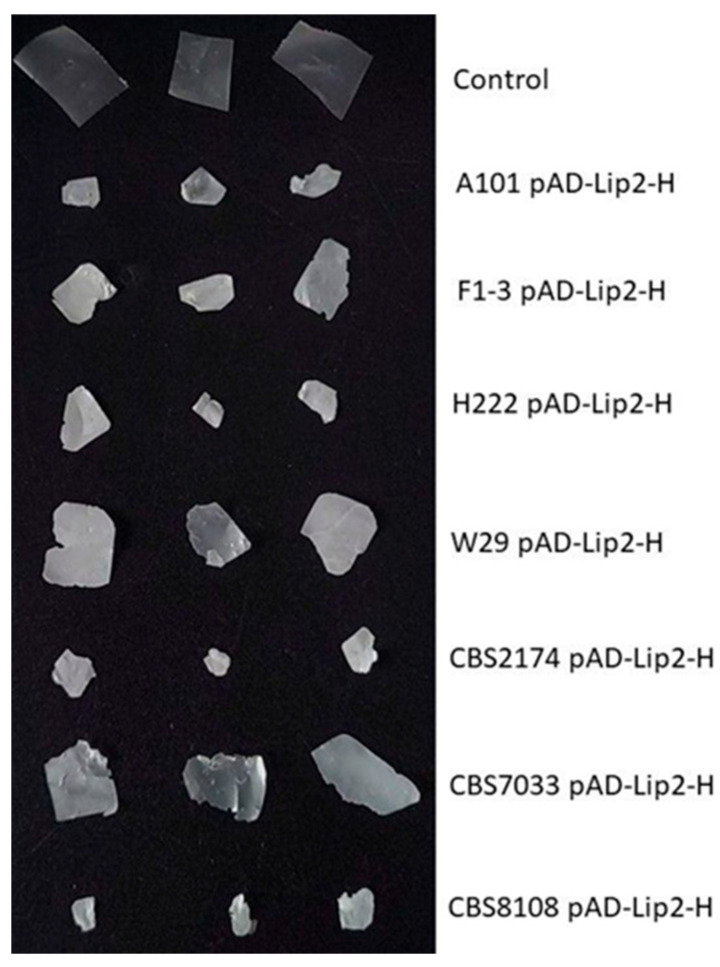
Macroscopic pictures of PCL film degradation after 24 h of incubation with the engineered *Y. lipolytica* strains overexpressing LIP2. As a control, PCL films incubated in YPD medium without any microorganisms were used.

In order to verify the ability of the engineered strains to degrade aliphatic polyesters, shake-flask cultures were carried out with the addition of PCL films. To this end, overnight cultures were grown in YPD medium; next, they were used as an inoculum to the shake-flask cultures in the baffled flasks. It was shown that *Y. lipolytica* requires a high concentration of dissolved oxygen (DO) in the medium [17], and employing baffled flasks improves protein production and enhances process productivity [18]. Already, after 24 h of the cultivation, we observed a fragmentation of the films. The films lost their original continuity and elasticity, breaking down into numerous small fragments with irregular edges (Figure 4). Employing the baffled flask resulted in a reduction in processing time because, in the previous study, this process took 144 h [5]. In agreement with the spot-test, we observed a difference between the strains (Figure S1). The least visible damage of PCL was observed for strain CBS7033 pAD-Lip2-H, whereas the most fragmented film had been found in the culture of CBS8108 pAD-Lip2-H, which clearly corresponds with qRT-PCR analysis (Figure 1) and the spot-test (Figure 3). Such changes in the macroscopic structure indicate deep changes in the polyester structure, leading to a loss of the material’s mechanical properties.

### 2.6. Scanning Electron Microscopy (SEM)

**Figure 5:**
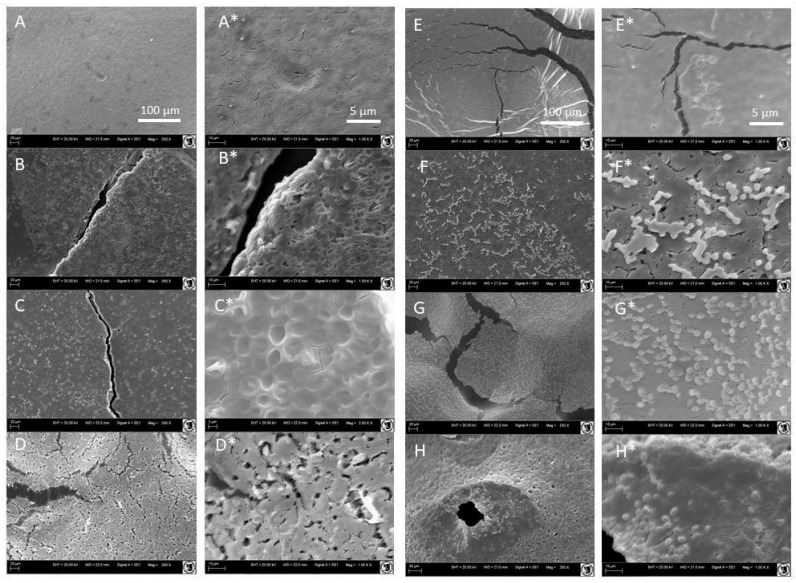
SEM images of PCL films recovered from shake-flask cultures after 24 h of incubation at 28 °C: (**A**,A*) control PCL film in YPD medium; (**B**,B*) A101 pAD-Lip2-H; (**C**,C*) F1-3 pAD-Lip2-H; (**D**,D*) H222 pAD-Lip2-H; (**E**,E*) W29 pAD-Lip2-H; (**F**,F*) CBS2174; (**G**,G*) CBS7033 pAD-Lip2-H; (**H**,H*) CBS8108 pAD-Lip2-H. Magnification of 250× (left panels) and 1000× (*, right panels).

Scanning electron microscopy was performed to characterize the surface changes of PCL after incubation in the shake-flask cultures of the engineered strains. As a control, we used films incubated in medium without any microorganism to check if mechanical forces have an impact on the surface of the films. As seen in Figure 5, the surfaces of PCL films after 24 h of incubation in YPD medium in the presence of different engineered strains looks differently for each strain. Depending on the strain, we observed cracks, tears and holes. For most of the strains, yeast cells were observed on the PCL films, with exceptions for strains H222 pAD-Lip2-H and W29 pAD-Lip2-H. Strain CBS2074 pAD-Lip2-H seems to produce a biofilm, since an additional surface on the yeast cell was observed (Figure 5F*).

## 3. Discussion

The growing accumulation of plastic waste has necessitated the development of efficient biotechnological recycling methods. While *Y. lipolytica* is an established platform for enzyme production, most of the studies are limited to a narrow group of model strains, such as W29 [14]. Moreover, study on PET biodegradation is mainly based on the heterologous expression of fungal cutinases and work on the purified enzyme [19]. In this study, we addressed this limitation by evaluating the influence of the genetic background of different wild-type isolates on the biodegradation of aliphatic polyester (PCL) through the overexpression of the native lipase LIP2.

The results show that the native LIP2 lipase has significant potential for the depolymerization of polyesters but its efficiency depends on the background. Wild-type strains showed negligible basal activity towards PCL, which is consistent with the strict regulation of the *Lip2* promoter by fatty acids and repressors in standard media [1]. Metabolic engineering allowed for the deregulation of this pathway, resulting in efficient enzyme secretion and the formation of distinct hydrolysis zones. Despite the fact that all strains belong to *Y. lipolytica* species, we observed significant differences between the wild types but also between the engineered strains. Interestingly, the most active strain was CBS8108, which was isolated from kerosene aviation fuel. This is clear evidence that yeast can adapt to hydrophobic substrates over time. This finding suggests that it is crucial to test several *Yarrowia* strains in order to select the optimal host for the further biodegradation process. The high level of native enzyme production did not appear to impose a metabolic burden on the host cells. The engineered strains maintained high growth rates and biomass, confirming that *Y. lipolytica* is a robust platform for whole-cell bioremediation processes. Although native lipases exhibit lower specific activity towards solid polyesters compared to fungal cutinases, this work confirms the potential of the native enzymatic machinery of *Y. lipolytica*. This offers a promising, self-sufficient platform that can be further optimized.

Unlike cutinases, which expose the active site, LIP2 from *Y. lipolytica* has a mobile element called a “lid” domain covering the catalytic triad [20]. This requires interfacial activation to expose the active site, which may limit the rate of hydrolysis on solid polymer surfaces compared to lidless cutinases. Nevertheless, SEM analysis confirmed that LIP2 effectively initiates surface erosion. Importantly, growth kinetics showed that high lipase production did not constitute a metabolic burden; engineered strains achieved high biomass densities, confirming the suitability of *Y. lipolytica* for whole-cell bioremediation applications. An undeniable advantage of the developed system is the rapid rate of biodegradation achieved under mesophilic conditions (28 °C). The visible fragmentation of PCL film was observed after only 24 h of incubation (Figure 4), which is a significant improvement compared to previous studies. For example, Ref. [5] showed that *Y. lipolytica* strains expressing heterologous cutinases required as long as 144 h to achieve a comparable degree of film degradation. It was shown that *Y. lipolytica* requires a high concentration of dissolved oxygen (DO) in the medium [17], and employing baffled flasks improves protein production and enhances process productivity. The reduction of the process time to 24–48 h in our study, achieved by overexpressing the native LIP2 in efficient wild-type strains (such as CBS8108), indicates a highly effective synergy between the host’s secretory capabilities and enzyme activity. Furthermore, the process is conducted at 28 °C, offering significant economic and environmental benefits compared to systems based on thermophilic microorganisms or enzymes. Many efficient polyester-degrading enzymes, such as cutinases from *Thermobifida* or *Thermomyces* species, require elevated temperatures (50–60 °C) to achieve optimal activity [3]. Although effective, such conditions generate high energy costs for heating, limiting their large-scale application. In contrast, our whole-cell system operates efficiently at 28 °C, drastically reducing the energy footprint. Additionally, compared to degradation by environmental fungal consortia (e.g., *Aspergillus* sp. or *Penicillium* sp.), which typically require weeks or months to degrade PCL in soil or compost [21], engineered strains of *Y. lipolytica* provide a fast and controllable solution for plastic waste disposal.

## 4. Materials and Methods

### 4.1. Microorganisms, Media, and Culture Conditions

The strains used in this study are listed in Table 1. YPD medium (1% yeast extract, 2% peptone, 2% glucose, 2% agar (only for solid medium), A&A Biotechnology, Gdańsk, Poland) was used for inoculum preparation and the routine passage of the strains. The bacterial strain Escherichia coli DH5α was used in plasmid cloning. The bacteria were grown in LB medium (1% tryptone, 0.5% yeast extract, 1% NaCl, A&A Biotechnology, Poland) with the addition of ampicillin (100 µg/mL, Sigma, Darmstadt, Germany). YPD medium with the addition of hygromycin at a concentration of 250 µg/mL was used for the selection of yeast transformants.

### 4.2. Genetic Engineering and Vector Construction

All restriction enzymes were purchased from FastDigest Thermo Scientific™ (Waltham, MA, USA), and all of the digestions were performed according to standard protocols. The PCR reactions were set up using recommended conditions and Phusion high-fidelity DNA polymerase (Thermo Scientific™, Waltham, MA, USA ). The ligation reactions were performed for 10 min at room temperature using T4 DNA Ligase (Thermo Scientific™). The *E. coli* minipreps were performed using the Plasmid Mini Kit (A&A Biotechnology, Gdańsk, Poland). The transformation of *E. coli* strains was performed using standard chemical protocols. Genomic DNA (gDNA) was extracted from *Y. lipolytica* using the Genomic Mini AX Yeast Spin kit (A&A Biotechnology, Gdańsk, Poland).

First, plasmid pAD-Hgr was formed. To this end, hygromycin-resistant cassettes have been amplified from vector pCfB5219, using primers Hgr-F and HGR-R (5′-CATGATATCTATGCTATACGAAGTTATAAG-3′) and (5′-GCGGATATCTGAATTCGGACACGGGCATCT-3′), creating 2481 bps PCR product. Next, pAD vector [18] was digested with *Eco32I*, and 7461 bp backbone was cut out from the gel. Vector and digested PCR product (with *Eco32I*) were ligated using T4 ligase and transformed into E. coli DH5α using standard procedures, resulting in pAD-Hgr. After the amplification of *Y. lipolytica* DNA with primers PR-Lip2-SgsI-F (5′-TAAGGCGCGCCATGAAGCTTTCCACCATCCT-3′) and PR-Lip2-Eco72I-R (5′-CGGCACGTGGCTTAGATACC ACAGACA-3′), gene encoding Lip2 (YALI0A20350g) was digested and cloned into the corresponding sites of pAD-Hgr, resulting in pAD-Lip2-H.

### 4.3. Transformation of Yarrowia lipolytica

The obtained pAD-Lip2-H plasmid was linearized with the MssI enzyme. The transformation of *Y. lipolytica* strains was performed using the lithium acetate method [2]. The transformation mixture contained competent yeast cells, carrier DNA (DNA from salmon sperm) and linearized plasmid. The mixture was seeded on YPD plates containing hygromycin. The plates were incubated at 28 °C until the appearance of transformant colonies (approx. 2–3 days). The proper integration of the cassettes was checked by PCR, as described before [18].

### 4.4. RNA Isolation and qRT-PCR

The shake-flask cultures were grown for 24 h in YPD. Next, the cultures were collected and centrifuged for 5 min at 12,000× *g*. The RNA was extracted using a Total RNA Mini Plus kit (A&A Biotechnology, Poland). Each sample was treated with DNAse I (Thermo Scientific™, Waltham, MA, USA) according to the manufacturer’s instructions, and the samples were stored in a −80 °C freezer. We conducted cDNA synthesis using Maxima First Strand cDNA. Synthesis kits for RT-qPCR (Thermo Scientific™, Waltham, MA, USA) were used according to the manufacturer’s instructions. We carried out qRT-PCR analyses using a DyNAmo Flash SYBR Green qPCR Kit (Thermo Scientific™, Waltham, MA, USA) and the CFX96 Touch Real-Time PCR Detection System (Bio-Rad Laboratories, Inc.). Primers for RT-PCR were designed as follows: gene qLip2-F (5′-AGCTTTCCACCATCCTCTTC-3′) and qLipd2-R (5′-TACACTCGCTTCTGGAGAAC-3′). The results were normalized to the actin gene ACT-F (5′-GAGTCACCGGTATCGTTC-3′) and ACT-R (5′-GCGGAGTTGGTGA AAGAG-3′) and analyzed using the ΔΔCT method [26]. Samples were analyzed in triplicate.

### 4.5. Growth Kinetics

The growth characteristics of the strains were determined in 96-well plates using a Spark reader (Tecan Group Ltd., Männedorf, Switzerland). A standardized cell suspension (OD_600_ = 0.1) was incubated in 200 µL of YPD medium at 28 °C for 72 h with vigorous orbital shaking. Optical density (OD_600_) measurements were performed automatically every 30 min. The experiment was performed in three independent biological replicates.

### 4.6. Clear Zone Formation Method

A preliminary assessment of biodegradation capacity was performed by the clear zone method as follows: ON cultures were incubated in YPD medium, the optical density (OD_600_) was adjusted to 0.1, 2 µL of the cultures were plated onto YNB medium with 0.1% emulsified polyesters (PCL) as described before [21], and the plates were incubated at 28 °C. After 24 h, the development of clear zones was observed.

### 4.7. Biodegradation Studies in Shake-Flask Culture

PCL degradation was performed in the 250 mL baffled flasks. PCL film was prepared by dissolving polycaprolactone in dichloromethane, pouring it onto Petri dishes, and evaporating the solvent. The dried films were cut into pieces (approx. 500 mg) and sterilized by rinsing in 70% ethanol and UV irradiation (10 min). The cultures were grown in 30 mL of YPD medium with 5% total glucose concentration. The inoculum was a 72 h preliminary culture, and the initial OD in the experimental flasks was set at 0.5. The control consisted of flasks with medium and film, without the addition of yeast. The cultures were incubated at 28 °C (200 rpm) for 48 h. After the culture was completed, the films were removed, thoroughly washed with distilled water, and dried, and their pictures were taken.

### 4.8. Surface Changes

Morphological changes in the surface of polycaprolactone (PCL) films after the end of cultivation were subjected to a two-stage assessment. Macroscopic analysis was performed based on photographic documentation taken with a 32 MP digital camera, assessing the degree of fragmentation and physical disintegration of the material. A detailed analysis of the surface structure was performed using a scanning electron microscope (SEM).

### 4.9. Scanning Electron Microscope (SEM)

In order to verify the influence of the microorganism’s activity on the polymer plastic surface, a scanning electron microscope was used. The investigation was conducted on three biological repetitions for each strain bearing overexpressing cassette; as a control medium, PCL films incubated under the same conditions were used. Film samples were mounted on standard SEM stubs using conductive carbon adhesive tape. The sample surfaces were subsequently sputter-coated with a thin gold (Au) layer for 300 s, resulting in a coating thickness of up to 2 nm. The sputtering process was carried out using an Edwards Scancoat Six Sputter Coater (United Kingdom) to enhance surface conductivity and minimize charging effects during electron beam exposure. Microstructural and surface topography analyses were performed using a ZEISS EVO LS15 (ZEISS, Germany)operating at an accelerating voltage (EHT) of 20 kV. Imaging was conducted in high-vacuum mode using a secondary electron detector (SE1) to obtain detailed surface morphology and dedicated software. All SEM observations were carried out at the Laboratory of Electron Microscopy, Wrocław University of Environmental and Life Sciences, Poland.

## 5. Conclusions

The studies showed that the genetic background of the host strain is a key factor determining the efficiency of polyester biodegradation. The constitutive overexpression of the native *Lip2* gene was successfully achieved in all tested *Y. lipolytica* isolates; however, the magnitude of the effect varied considerably depending on the host strain. This finding highlights the importance of using various wild-type strains as a chassis for biotechnological processes, thereby exploiting the intrinsic potential of *Y. lipolytica*.

## Figures and Tables

**Table 1:** Strains and vectors used in this study.

Strain	Substrate of Isolation/Origin	References
***E.* *coli***		
DH5α	Cloning host, F- endA1 glnV44 thi-1 recA1 relA1 gyrA96 deoR nupG Φ80dlacZΔM15 Δ(lacZYA-argF)U169, hsdR17(rK− mK+), λ−	[22]
pAD	Vector with UAS_B16_-TEF promoter	[23]
pAD-Hgr	pAD with Hgr resistant cassette	This study
pAD-Lip2-H	pAD-Hgr with *Lip2* overexpressing cassette	This study
***Y. lipolytica***		
A101	Polluted soil at a car wash, Poland	[24]
F1-3	Cheese, France	[25]
H222	Soil, Germany	[25]
W29	Sewer of Paris, France	[25]
CBS2074	Olives, Italy	[25]
CBS7033	Soil, Japan	[25]
CBS8108	Kerosene aviation fuel	[25]
A101 pAD-Lip2-H	A101 harboring *Lip2* overexpressing cassette	This study
F1-3 pAD-Lip2-H	F1-3 harboring *Lip2* overexpressing cassette	This study
H222 pAD-Lip2-H	H222 harboring *Lip2* overexpressing cassette	This study
W29 pAD-Lip2-H	W29 harboring *Lip2* overexpressing cassette	This study
CBS2074 pAD-Lip2-H	CBS2074 harboring *Lip2* overexpressing cassette	This study
CBS7033 pAD-Lip2-H	CBS7033 harboring *Lip2* overexpressing cassette	This study
CBS8108 pAD-Lip2-H	CBS8108 harboring *Lip2* overexpressing cassette	This study

## Data Availability

The original contributions presented in this study are included in the article/Supplementary Materials. Further inquiries can be directed to the corresponding author.

## Supplementary Materials

The following supporting information can be downloaded at: https://www.mdpi.com/article/10.3390/ijms27094073/s1.
