# Supplementary material for: Yeast Yarrowia lipolytica as a Chassis for Polyester Biodegradation—A Comparative Analysis of the Diverse Wild-Type Strains and Their Engineered Derivative That Overexpresses the LIP2 Lipase
Source: Int J Mol Sci. 2026 May 1;27(9):4073. doi: 10.3390/ijms27094073 (PMC13163340; doi:10.3390/ijms27094073)
Supplement: Supplementary file 1 [file ijms-27-04073-s001.zip › ijms-4252181-supplementary.pdf]

International Journal of
Molecular Sciences

MDPI

Supplementary Material

Yeast *Yarrowia lipolytica* as a chassis for polyester biodegradation- a comparative analysis of the diverse wild-types strains and theirs engineered derivative overexpressing the LIP2 lipase

Julia A. Dybka and Aleksandra M. Mirończuk*

Laboratory for Biosustainability, Institute of Biology, Wrocław University of Environmental and Life Sciences, ul. Kożuchowska 5b, 51-630, Wroclaw, Poland
* Correspondence: aleksandra.mironczuk@upwr.edu.pl

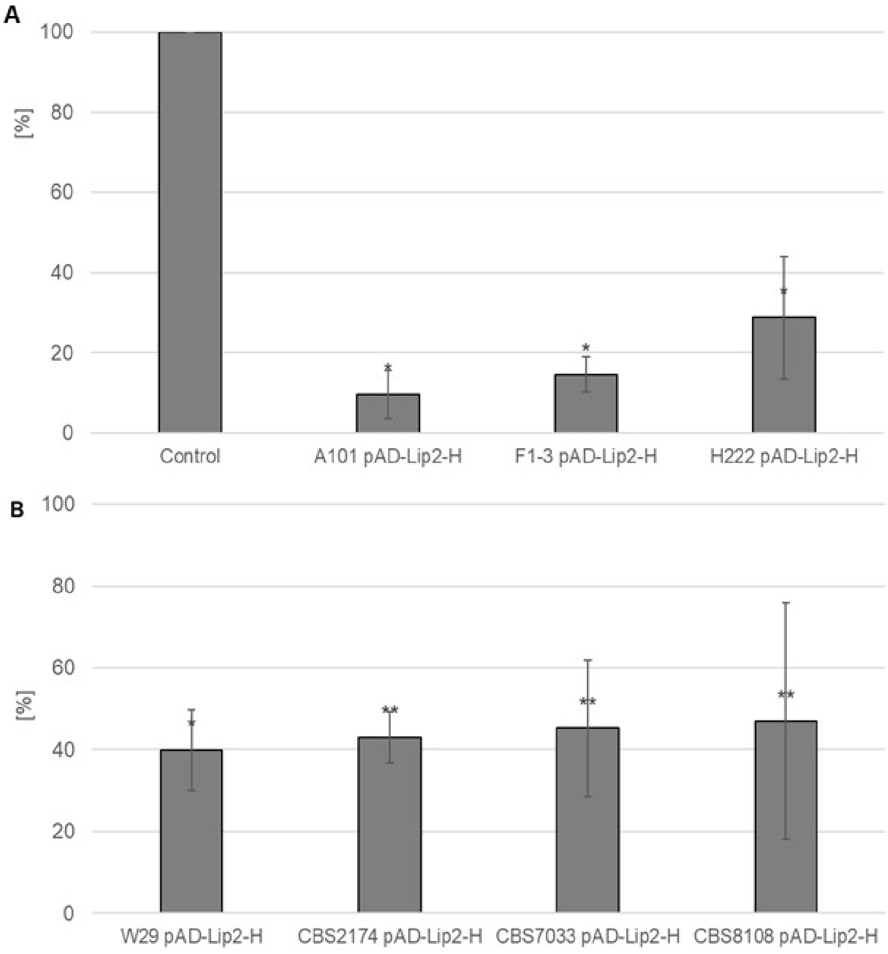

Figure S1. Remained weight (%) of the PCL films during 96 hours of cultivation of PCL film in conical flask with strains A101, F1-3, H222 (A) and W29, CBS2174, CBS7033 and CBS8108 (B) overexpressing pAD-Lip2-H. Cultivation was performed at 28 °C at constant agitation (200 RPM) in YPD medium. Three biological replication was used in this experiment. Error bars present SD.
